# Individual Cell Based Traits Obtained by Scanning Flow-Cytometry Show Selection by Biotic and Abiotic Environmental Factors during a Phytoplankton Spring Bloom

**DOI:** 10.1371/journal.pone.0071677

**Published:** 2013-08-12

**Authors:** Francesco Pomati, Nathan J. B. Kraft, Thomas Posch, Bettina Eugster, Jukka Jokela, Bas W. Ibelings

**Affiliations:** 1 Department of Aquatic Ecology, Swiss Federal Institute of Aquatic Science and Technology (Eawag), Dübendorf, Switzerland; 2 ETH-Zürich, Institute of Integrative Biology (IBZ), Department of Environmental Systems Science, Zürich, Switzerland; 3 Department of Biology, University of Maryland, College Park, Maryland, United States of America; 4 Limnological Station, Institute of Plant Biology, University of Zürich, Kilchberg, Switzerland; 5 Université de Genève, Institut F.-A. Forel, Versoix, Switzerland; Consiglio Nazionale delle Ricerche (CNR), Italy

## Abstract

In ecology and evolution, the primary challenge in understanding the processes that shape biodiversity is to assess the relationship between the phenotypic traits of organisms and the environment. Here we tested for selection on physio-morphological traits measured by scanning flow-cytometry at the individual level in phytoplankton communities under a temporally changing biotic and abiotic environment. Our aim was to study how high-frequency temporal changes in the environment influence biodiversity dynamics in a natural community. We focused on a spring bloom in Lake Zurich (Switzerland), characterized by rapid changes in phytoplankton, water conditions, nutrients and grazing (mainly mediated by herbivore ciliates). We described bloom dynamics in terms of taxonomic and trait-based diversity and found that diversity dynamics of trait-based groups were more pronounced than those of identified phytoplankton taxa. We characterized the linkage between measured phytoplankton traits, abiotic environmental factors and abundance of the main grazers and observed weak but significant correlations between changing abiotic and biotic conditions and measured size-related and fluorescence-related traits. We tested for deviations in observed community-wide distributions of focal traits from random patterns and found evidence for both clustering and even spacing of traits, occurring sporadically over the time series. Patterns were consistent with environmental filtering and phenotypic divergence under herbivore pressure, respectively. Size-related traits showed significant even spacing during the peak of herbivore abundance, suggesting that morphology-related traits were under selection from grazing. Pigment distribution within cells and colonies appeared instead to be associated with acclimation to temperature and water chemistry. We found support for trade-offs among grazing resistance and environmental tolerance traits, as well as for substantial periods of dynamics in which our measured traits were not under selection.

## Introduction

The study of trait-environment relationships is fundamental for understanding the evolution of phenotypic characters and ecological dynamics among species. Modern concepts of community ecology, for example, propose that the diversity of natural communities is driven by traits characterizing species strategies tolerating and exploiting the environment, differences in competitive ability or enemy-resistance traits, as well as by stochastic processes that are trait-neutral [1]–[5]. Trait-based approaches focus on the phenotypic traits of individuals, populations or species to understand structure and functioning of natural communities [6]–[8]. One aim of trait-based approaches is to offer a “common currency” by which to compare taxa, in order to illuminate general rules of community dynamics, in contrast to species-specific studies which often reveal idiosyncratic responses [9]. Trait-based approaches provide the opportunity to study factors that determine changes in community structure and functioning, or infer potential mechanisms of selection in complex communities by comparing observed trait patterns to predictions from theory [4], [6], [10]–[12] (Table 1).

**Table 1:** Predicted influence of selection processes on trait community structure, assuming that the community includes only one habitat.

	Trait patterns	
Process	Habitat-occupancy traits^a^	Species-interaction traits^b^
Environmental filtering	clustered	random
Competition	clustered	evenly dispersed
Grazing/predation	random	evenly dispersed or clustered

Random patterns are expected in all cases when focal traits are not under selection. Adapted from [4], [10], [14], [15].

a Environmental tolerance and resource use strategy traits: allow species to establish themselves and thrive in a community due to compatibility with environmental conditions and resources [4], [10]. In the case of this study, examples include type and cellular levels of active pigments [18].

b Enemy resistance or resource acquisition traits that provide an advantage over competitors, predators or parasites: they drive competitive exclusion through the ability of exploiting common limiting resources or convey resistance to grazers, predators and parasites [1], [4], [10]. In the case of this study, examples include size, coloniality, shape and cell volume [17], [18].

We can expect, for example, that when selection by abiotic environmental filters is in operation on a trait associated with habitat specificity, its community-wide distribution would converge on similar values (mean trait values of species would be shifting to the same direction), or filtering would reduce the range of the trait distribution [11], [13], [14] (Table 1). A clustered pattern in the distribution of habitat-related traits, however, can be also obtained when strong negative interactions (like competition) lead to species exclusion (e.g. reducing community trait range) [4] (Table 1). Conversely, when species interactions such as competition for resources or grazing/predation/parasitism from specialists dominate, we expect that species would show dissimilar enemy-resistance or resource-acquisition traits (dispersion of strategies), resulting in more evenly spaced traits along community trait axes [10], [11], [14], [15] (Table 1). Finally, we expect that generalist enemies or predators will cluster the community around selected species interaction traits or trait values [10], [15], [16]. When multiple selection processes such as environmental filtering and enemy mediated selection are co-occurring on a community, its structure may be explained by trade-offs between different ecological strategies mediated by environmental tolerance, resource acquisition and enemy resistance traits [10], [17], [18].

Here, we studied trait changes in a phytoplankton community during a spring bloom composed of a succession of environmental conditions, phytoplankton species and their grazers in Lake Zurich, Switzerland. Phytoplankton is a classical ecological model system with much knowledge about key ecological traits. Examples include cell size, shape and coloniality (which influence motility, grazing resistance, and nutrient uptake through surface area/volume ratio - SA/V), photosynthetic pigment type and concentration (which relates to photosynthetic performance and adaptation to different light environments), N_2_-fixation and mixotrophy (which define nutrient uptake strategies) [18]. Traits derived from laboratory studies appear to retain predictive power to explain the dynamics of natural populations [19], however empirical evidence of trait-environment relationships from phytoplankton under natural community dynamics in the field is lacking.

In this study we aimed at identifying relationships between traits and their environment during the phytoplankton spring bloom, and evaluating selection gradients caused by relevant interactions such as competition or grazing (Table 1) [20], [21]. The spring bloom is considered as a prime example of the importance of physio-morphological traits in shaping community dynamics. The succession starts with the buildup of a community of small celled phytoplankton taxa with a high SA/V, which benefit from ample nutrients and improving light conditions at the end of the winter [22]. This spring bloom increases the fecundity of zooplankton, which become abundant and graze down the phytoplankton. Next, grazing resistance is gained by large cell size or by colony formation, which both decrease nutrient competitive ability due to reduced SA/V, thus creating a possible trade-off that may allow species co-existence or turnover [22]–[24]. Most evidence for the role of physio-morphological traits in this generally well known ecological succession is, however, derived from theory or laboratory evidence [18], [25].

Our specific objectives were to investigate how phytoplankton groups and their expressed trait values respond to environmental selection before, during and after the grazing period. Natural communities pose methodological constraints on empirical work, including measuring traits accurately and at the relevant spatial and temporal scale. Scanning flow-cytometry allows to monitor - at the level of individual particles - a relatively large number of important physio-morphological features such as size, coloniality, pigment type and content, pigment distribution within cells or colonies in natural populations, and to classify phytoplankton into groups based on these measured traits [26]–[29]. Here we used the fourth corner method [30] to test for links between average population trait values derived by flow-cytometry, population abundance and environmental variables. The aim was to screen several traits and identify those that were under selection (shifts in mean values) over the entire period of study. This set of focal traits was then studied for their community wide trait patterns [14] at each day of the temporal series in relation to the hypothesized selection processes as outlined in Table 1.

## Materials and Methods

### Sample Collection and Limnological Methods

We sampled the peri-alpine mesotrophic Lake Zürich (Switzerland) from the 23^rd^ of March to the 6^th^ of May 2009 every 2 to 4 days at 10 a.m., offshore from Kilchberg (maximum depth 100 m, 47°19.3'N 8°33.9'E). No specific permission was required for sampling since the lake is a public water body, and the study did not involve any endangered or protected species. Eawag and the University of Zürich are Federal and Cantonal institutions, respectively, and have the mandate from the government to sample and monitor lakes in Switzerland. Profiles (0 to 40 m) of pressure (depth), oxygen, turbidity, temperature and conductivity were obtained using a 6600 YSI multiprobe (YSI Inc., Yellow Springs, OH, USA). Water samples were taken with a five liter sampler (Uwitec, Mondsee, Austria) at the depth of maximum chlorophyll-a (Chl-a) concentration, measured on site over the water column with a fluoroprobe (TS-16-12, bbe-Moldaenke GmbH, Kiel, Germany). This depth reflected the area of maximum productivity and biomass for the plankton community. Abundance of ciliates was determined via Protargol-staining of fixed samples (300 mL), while plankton net (mesh size 30 µm) live samples were used for taxonomic determination [31]. Dissolved organic carbon (DOC), dissolved reactive phosphorous (P-PO4) and nitrate (N-NO3) were measured on collected water samples using standard methods [32]. We determined and counted phytoplankton taxa using inverted microscopy from 100 mL Lugol-acetic preserved samples. For flow-cytometry analysis, 50 mL of sampled water were fixed with a filter-sterilized solution of paraformaldehyde and glutaraldehyde (0.01 and 0.1% final concentration, pH 7) and stored at 4°C in the dark. Macro-zooplankton was sampled monthly during the routine monitoring campaign of Lake Zurich in the center of the lake (in front of Thalwil, circa 3.5 Km from the Kilchberg sampling site), by net-collection (mesh size 95 µm) from bottom (136 m) to top (0 m).

### Flow-cytometry

We used a scanning flow-cytometer from Cytobuoy (Woerden, the Netherlands; http://www.cytobuoy.com) for counting, characterization and classification of phytoplankton [27]–[29], [33]. This instrument is designed to analyze the naturally occurring size range from small (e.g. picoplankton) to large (e.g. colonial) plankton species (0.5 to 900 µm in diameter and a few mm in length). Each particle was intercepted by a coherent solid-state Sapphire 488 nm laser beam (15 mW) at the speed of 2 m s^−1^. More details on the instrument can be found elsewhere [27]. Digital data acquisition was triggered by the sideward scatter (SWS) signal with a trigger-level of 20 mV, which excludes particles smaller than 0.5 µm. The light scattered (908 nm) from each passing particle was measured at two angles, forward scatter (FWS) and SWS, to provide information on size and shape of particles. The fluorescence (FL) emitted by photosynthetic pigments in algal cells was detected at three different wavelengths: red (FLR), orange (FLO) and yellow (FLY) signals were collected in ranges of 668–734 (Chl-a), 601–668 (phycocyanin and phycoerythrin) and 536–601 nm (decaying pigments), respectively [34]. Laser alignment and calibration were done before analysis using yellow fluorescence beads of 1 and 4 µm. In this study, we scanned roughly 30,000 particles for each sample.

### Data Preparation and Clustering of Phytoplankton Particles

Data manipulation, analysis and graphics were performed with the R statistical programming language [35]. The Cytobuoy allows the analysis of pulse-signals providing, in the structural configuration used for this study, 45 descriptors of 3D structure and FL profile for each particle [33]. Raw Cytobuoy data were visually inspected for the distribution of FL signals in order to set threshold levels to extract FL particles (phytoplankton) with a size larger than 2 µm. Phytoplankton concentrations were calculated by inferring the number of cells from the number of humps in the SWS signal of each particle to account for colonial species [34]. Phytoplankton biovolumes were estimated for each particle assuming an ellipsoid shape and based on Total FWS signal using the formula Biovolume^2^ = 0.0017 Total.FWS –0.013 [29], [36]. Cytobuoy particle descriptors are expressed in different units and some are cross-correlated (data not shown). Particle descriptors were therefore standardized and, by principal component analysis (PCA), reduced to 28 orthogonal vectors covering 99% of the total variance in the data (for factor loadings of PCA see Table S1).

Different approaches have been previously used in phytoplankton to organize species into categories based on ecological and functional characteristics [37], [38], on purely morphological traits [39], and on both ecological and morphological characters [40]. In this study we relied on unsupervised model-based clustering to group phytoplankton, applying maximum likelihood estimation and Bayesian criteria to identify the most likely model and number of clusters, using principal components (PCs) of Cytobuoy particle descriptors [29]. Specifically, we used the R package *mclust* in which the optimal model is selected according to Bayesian Information Criterion initialized by hierarchical clustering for parameterized Gaussian mixture models [41]. To accommodate for computational issues, we limited the final clustering dataset to 10,000 phytoplankton particles (out of a total of 25,000), randomly extracted from the database. The optimal model in our study corresponded to 22 ellipsoidal, equally shaped clusters. For the determination of presence/absence of cytometry-derived groups, we applied a concentration threshold (1 cell/mL) below which a group was considered as virtually not present. This threshold (and the cut-off of particles smaller than 2 µm) was implemented for comparison of flow-cytometry data with microscopic counts since picoplankton and small nano-phytoplankton, in particular, are commonly not efficiently counted by microscopy when rare [42].

### Linking Flow-cytometry Derived Traits to the Environment

We used the two-step fourth corner method [30] to test the correlation between Cytobuoy-derived phytoplankton traits and environmental variables, weighed by the abundances of phytoplankton clusters. This method allows to test for selection by environmental filters on focal traits by statistically linking a species abundance matrix (L, abundance for phytoplankton trait-based groups at different days in this study), with a matrix (R) consisting of the environmental variables of the sites and a matrix (Q) containing average trait values for each group [30]. Our trait matrix included selected flow-cytometry signal parameters and the first axis of the PCA (40% of variance in the data, Table S1). The link between quantitative phytoplankton traits and environmental variables was measured by a Pearson correlation coefficient and the significance was tested by comparing results of two permutation models (999 iterations): model A (permutes values of days, i.e. rows of L) and model B (permutes values of species, i.e. columns of L) [30]. Model A tests H_0_ (no link between R, L and Q) against H_1_ (L and R are linked), and model B tests H_0_ against H_2_ (L and Q are linked). Combining results allows a test of H_0_ against H_3_ (matrices R, L, and Q are linked): the significance of the H_3_ test (final p-value) was extracted as the maximum of individual p-values for H_1_ and H_2_ tests [43]. We computed the autocorrelation function for all our variables to assess serial autocorrelation in our data, finding weak signs of temporal autocorrelation. To test for potential interference of temporal autocorrelation in our analysis, we included the series of sampling dates as one of the environmental variables in the fourth-corner method finding no significant results (data not shown).

### Linking Trait Distributions to Selection Processes

To detect non-random patterns in phytoplankton community-wide trait distributions over each day of our time-series, we applied trait-based community tests using flow-cytometry derived groups of phytoplankton [11], [14]. Trait means for each phytoplankton group were matched to the groups present in each day of the series to calculate a community-wide frequency distribution of trait values. For this analysis we focused on three phytoplankton traits: length by SWS (particle size), Fill factor (pigment distribution within particle) for FLR (Chl-a) and FLO (phycocyanin). We chose these traits because of their possible influence on motility, grazing resistance, pigment strategy and acclimation to light and water chemistry. Length and FLR were Log_10_ transformed in order to account for non-normal distribution. We also included PC1 (accounting for circa 40% of total multivariate trait variance) as an aggregated index of phytoplankton morphology (Table S1). SWS length, Fill factor FLR and FLO loads on PC1 were 0.172, −0.08 and −0.09, respectively (Table S1).

We used community trait mean and range, and the standard deviation of successive neighbor distances along trait axes divided by range (SDNDr), to measure the effects of habitat filtering and species interactions on our focal traits (Table 1) [11], [13], [14]. SDNDr quantifies how regularly spaced the trait-based groups are across a given range of trait values [14] (Table 1). For each day, the observed metrics were compared to a null expectation generated by creating 999 random communities of equal richness by drawing groups from the entire time-series weighted by their series-wide frequency of occurrence (the fraction of days in which each group was present), irrespective of trait values [14]. As a means of assessing the significance of each metric, we judged each datum as significantly non-random if the observed metric fell into the extreme 5% of the null distribution for that day of the time-series. One-tailed and two-tailed tests were used to assess changes in mean values and in the other metrics, respectively [11], [14].

## Results

### Spring Bloom Dynamics in Lake Zürich

**Figure 1:**
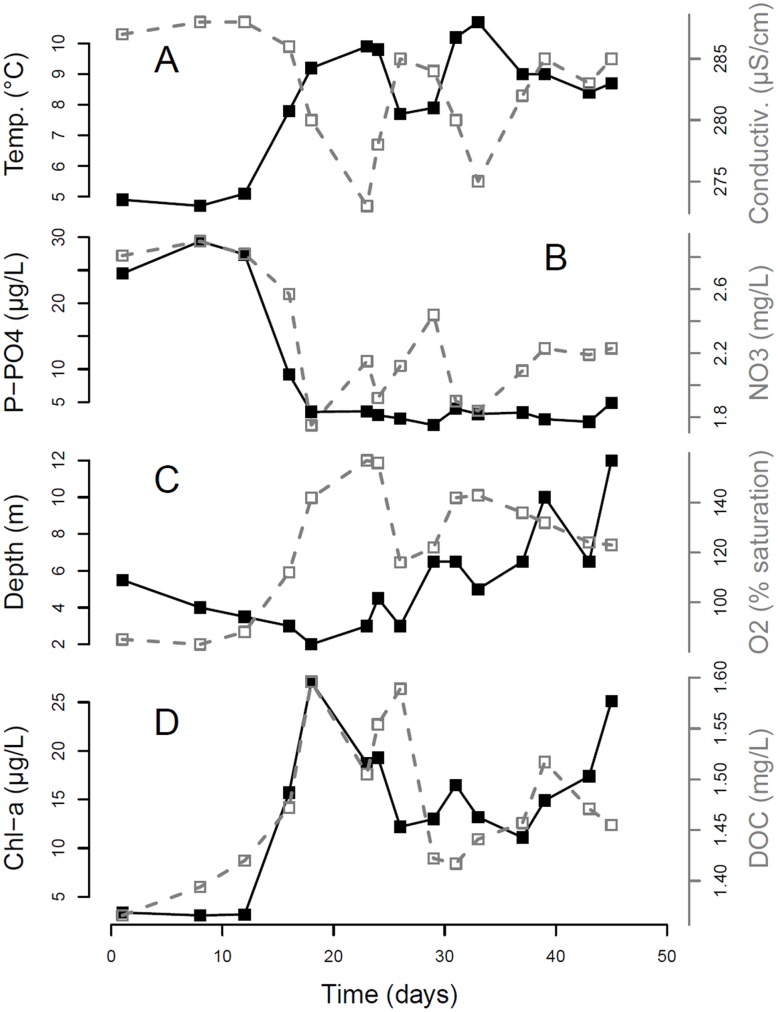
Spring bloom dynamics in Lake Zurich over the period of study (45 days between March 23 and May 6, 2009). (A) water temperature (black solid line) and conductivity (grey dashed line); (B) free available phosphorus (black solid line) and nitrates (grey dashed line); (C) depth of the Chl-a maximum (the sampled community, black solid line) and oxygen levels (grey dashed line); (D) Chl-a concentration (black solid line) and dissolved organic carbon (grey dashed line).

We observed the onset of phytoplankton growth when the surface water temperature reached 7°C (the 7^th^ of April - day 15 of our study period, Fig. 1A). The fast and intense spring phytoplankton proliferation caused a rapid decrease in dissolved nutrients (Fig. 1B). The bloom was initially characterized by a shallow Chl-a maximum (2 to 3 meters), which increased in magnitude and depth along with the progression of bloom dynamics (Fig. 1C). The peak of maximum phytoplankton productivity was reached between day 18 (9^th^ of April) and 24 (15^th^ of April) of the series (Fig. 1C–D). At the depth of the Chl-a maximum, the spring bloom was characterized by a succession of taxa belonging to the classes *Cryptophyceae*, *Chrysophyceae* and *Bacillariophyceae* over a 15 day time interval (from the 7th to the 22^nd^ of April). This was followed by a period of dominance by the cyanobacterium *Planktothrix rubescens* (Table S2), which accounted for 92% of all phytoplankton cells (Table S2) in the community characterized by a metalimnetic Chl-a maximum.

**Figure 2:**
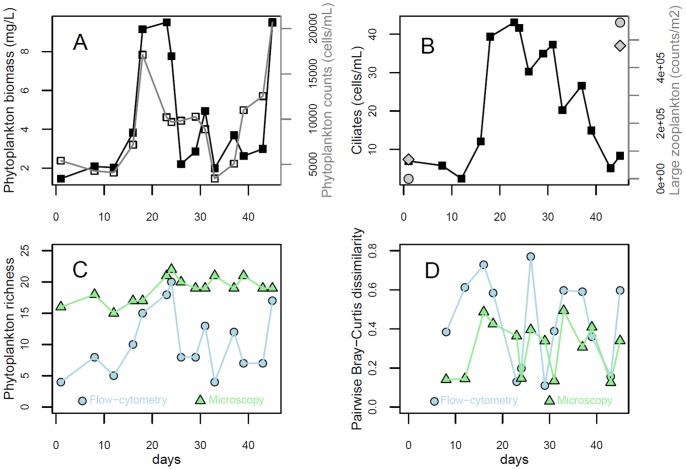
Spring bloom dynamics in Lake Zurich over the period of study (45 days between March 23 and May 6, 2009) at the depth of Chl-a maximum (Fig. 1). (A) Total biovolume of phytoplankton cells measured by flow-cytometry (black solid line) and phytoplankton concentration obtained by microscopic counts (grey line). (B) Concentration of pelagic herbivore ciliates (black line) and counts for Cladocerans (○) and Copepods (⋄). Richness (C) and pairwise Bray-Curtis dissimilarity (calculated for adjacent time points, D) of phytoplankton groups derived by flow-cytometry analysis compared to taxonomic groups obtained by microscopy.

Phytoplankton abundance and biovolume peaked between day 16 and 26 (between the 7^th^ and the 17^th^ of April) and at the end of the time series (Fig. 2A). The spring phytoplankton bloom was coupled to a parallel increase in the density of herbivorous planktonic ciliates (Fig. 2B). These were mainly taxa of the orders *Oligotrichida* and *Prostomatida*, which preferentially prey on Cryptomonads and small diatoms. The density of herbivorous ciliates showed a peak just after phytoplankton reached the maximum abundance on day 23 of the time series, and phytoplankton density concomitantly declined (Fig. 2B). Meso- and macro-zooplankton (Copepoda and Cladocera), which increased in abundance in May-June, may have been responsible for grazing at the end of the spring bloom succession (Fig. 2B).

The peak of phytoplankton biomass and herbivore ciliate density was associated with the highest phytoplankton richness at the depth of maximum Chl-a, both in terms of species and flow-cytometry derived groups (Fig. 2C). The number of phytoplankton taxa was higher than flow-cytometry derived groups throughout the bloom and grazing period, during which they reached a maximum of 22 and 20 units, respectively, at maximum. After the period of intense grazing, the dynamics of phytoplankton taxa and trait-based groups appeared to be slightly de-coupled, with larger numbers of taxa associated with lower levels of trait-based richness (Fig. 2C). Patterns of turnover of phytoplankton taxa and trait-based groups, assessed as pairwise measurements of Bray-Curtis dissimilarity among adjacent time points, showed that microscopic and flow-cytometry based analyzes captured similar trends, with the dynamics of trait-based groups being more pronounced (Fig. 2D).

### Trait-environment Relationships

Overall, trait-environment relationships assessed in this study appeared to be rather weak (Table 2 and Table S3), with Pearson’s correlation values ranging from –0.181 to +0.166. Measured phytoplankton traits appeared to correlate more significantly with conductivity, oxygen and temperature (Tables 2 and S3). PC1 (an aggregated descriptor of particle morphology, Table S1), number of cells per colony, total (integrated) and maximum levels of FL signals all directly co-varied with the size (length) of phytoplankton particles (data not shown) and therefore responded similarly to environmental variables with regards to the significance of the relationship, albeit with slightly different correlation coefficients (Table 2). These size-related traits showed a positive correlation with temperature and oxygen levels, and a negative correlation with conductivity (Table 2). The Fill factor of FL signals (within particle distribution of pigments) showed opposite relationships to environmental conditions compared to size-related traits (Table 2). No significant linkage between traits and nutrients was found at α = 0.05. For α = 0.1, the Fill factor of FL signals appeared to be weakly and positively linked to free available PO4 (Table S3). The Fill factor of orange (phycocyanin) and yellow (decaying pigments) fluorescence also showed to be negatively related to the abundance of herbivore ciliates in the community (Table 2).

**Table 2:** Results of the two-step fourth corner analysis (Pearson’s correlation coefficients) performed on Cytobuoy-derived phytoplankton traits and environmental variables.

Cytobuoy-derived traits	Temperature	Conductivity	Oxygen %	DOC	PO4	NO3	Ciliates
PC1^a^	**0.135**	**−0.172**	**0.154**	0.044	−0.081	−0.068	0.112
Length.SWS^b^	**0.132**	**−0.172**	**0.149**	0.040	−0.076	−0.061	0.101
Total.FL.Yellow^c^	0.104	**−0.140**	0.119	0.029	−0.059	−0.041	0.074
Total.FL.Orange^c^	0.076	−0.113	0.091	0.025	−0.043	−0.022	0.050
Total.FL.Red^c^	0.098	**−0.135**	0.114	0.030	−0.056	−0.037	0.070
Max.FL.Yellow^d^	0.130	**−0.130**	0.132	0.027	−0.080	−0.075	0.102
Max.FL.Orange^d^	0.130	**−0.156**	**0.143**	0.034	−0.075	−0.060	0.099
Max.FL.Red^d^	0.134	**−0.132**	0.140	0.043	−0.096	−0.086	0.120
Fill.FL.Yellow^e^	−0.151	**0.153**	**−0.164**	−0.066	0.114	0.113	**−0.161**
Fill.FL.Orange^e^	**−0.148**	**0.138**	**−0.157**	−0.065	0.114	0.106	**−0.148**
Fill.FL.Red^e^	−0.125	0.122	−0.140	−0.065	0.108	0.102	−0.133
Num.Cells.SWS^f^	**0.153**	**−0.181**	**0.167**	0.048	0.092	−0.081	0.118
Num.Peaks.FL.Yellow^f^	0.125	**−0.169**	**0.142**	0.032	0.059	0.062	0.097
Num. Peaks.FL.Orange^f^	0.122	**−0.164**	0.136	0.028	−0.056	−0.059	0.091
Num. Peaks.FL.Red^f^	0.130	**−0.159**	0.141	0.038	−0.067	−0.062	0.100

Significant correlations at *p*>0.05 are highlighted in bold (n = 22-groups × 15-sites × 15-traits). Corresponding p-values are reported in Table S3.

a First principal component of all traits, aggregated representation of phytoplankton morphology (see Table S1);

b Length by SWS, the most accurate measure of particle length;

c Total FL (integrated signal),

d Maximum amplitude of FL;

e Fill factor, between 0 and 1, gives information on how the signal resembles a square signal, i.e. very low values indicate that the signal is concentrated in a very narrow peak, indicative of an uneven distribution of pigments within cell/colony;

f number of humps in the signal, proportional to number of cells for colonial phytoplankton or the number of FL peaks per particle.

### Patterns in Community Wide Trait-distributions

We chose four phytoplankton descriptors as representative for size- and FL-related traits: length by SWS, PC1, Fill factor for Chl-a (Fill.FLR) and phycocyanin (Fill.FLO). To assess deviations from random patterns in their distributions in the phytoplankton community, we considered three distinct phases in the period of study: (1) before start of the spring bloom (day 1 to 16, between the 23^rd^ of March and the 7^th^ of April); (2) the actual spring bloom succession of producers and their grazers (day 18 to 31, between the 9^th^ and the 22^nd^ of April); (3) the period after the bloom characterized by the outcome of dynamic community interactions from phase 2 and the dominance of *P. rubescens* (day 33 to 45, between the 24^th^ of April and the 6^th^ of May).

**Figure 3:**
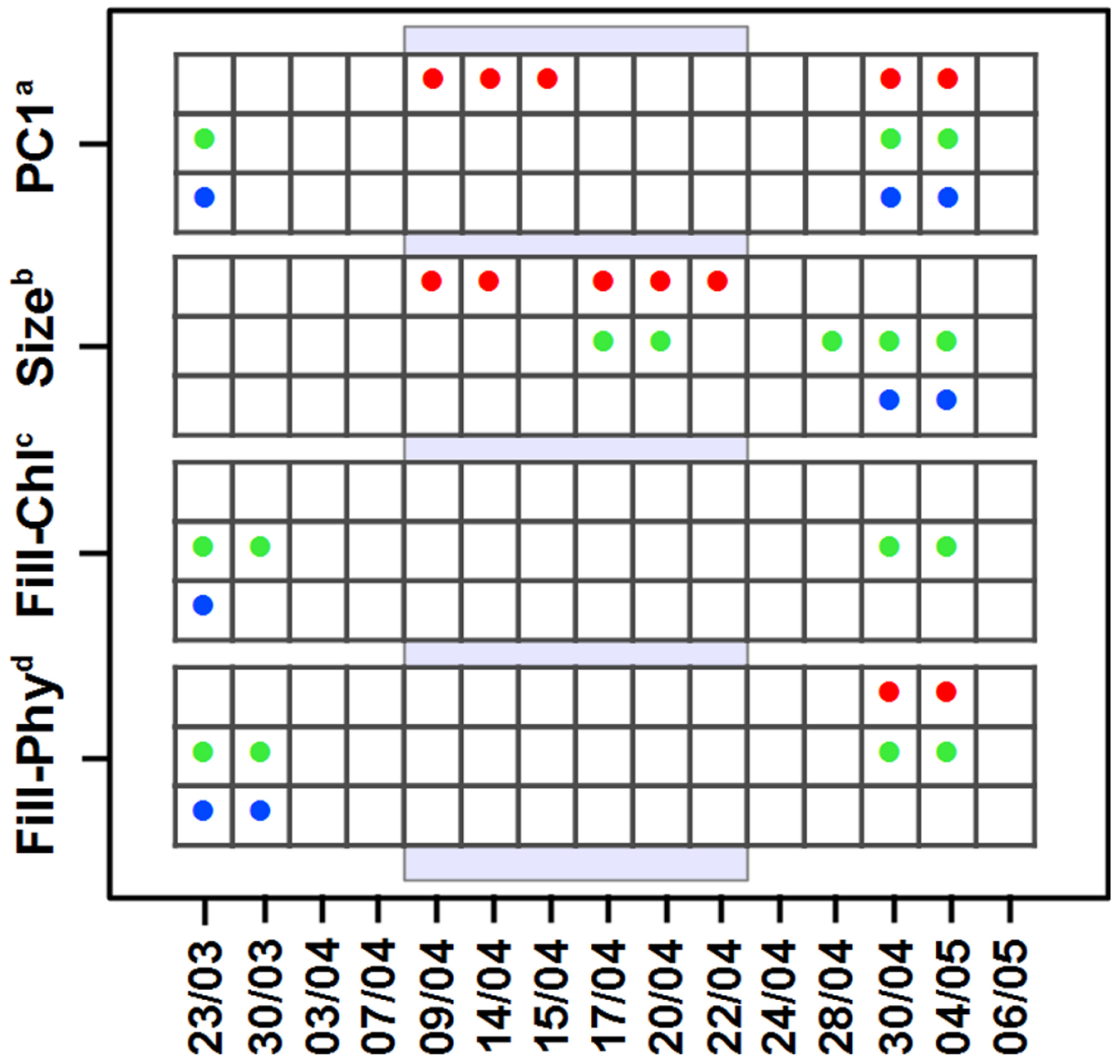
Summary of trait-based tests for community assembly at each sampling date of our study period: a) first principal component of Cytobuoy-derived phytoplankton traits (Table S1); b) size of phytoplankton particles; c) Chl-a particle fill; d) phycocyanin particle Fill (see Methods and Table 2). Dots indicate statistically significant deviations from null-model expectations (Wilcoxon p-value >0.05): red = SDNDr (even spacing of traits); green and blue = distribution range and mean, respectively (environmental filtering). The grey shaded area emphasizes the period of herbivore ciliate grazing (phase 2 in the text).

Before the spring bloom and the period of grazing (phase 1), Chl-a and phycocyanin Fill factors showed statistically significant evidence for a shift in mean values, and a reduced range compared to the null-model expectation in the first and second days of our time-series (Fig. 3 and Table S4–S5). This initial period, characterized mainly by low algal proliferation, appeared to be associated with phytoplankton size distributions that were not distinguishable from null-model expectations (Fig. 3 and Tables S4–S5). PC1, a multivariate phytoplankton descriptor that has both size, shape, and FL related components, showed significant signals of environmental filtering in day 1 (Fig. 3).

The second phase was characterized by significant evidence for even spacing of PC1 and size of phytoplankton particles (Fig. 3). In the phytoplankton size distributions, we also found evidence for reduced range during herbivore grazing (Fig. 3). During grazing, community-wide distributions in Chl-a and phycocyanin Fill factors were not significantly different from random expectations (Fig. 3).

After the peak in density of ciliate herbivores (phase 3), all the chosen traits showed some significant reduction in range within the community, with PC1 and size also displaying shifts in mean values comparing to null model expectations (30^th^ of April and 4^th^ of May, Fig. 3). At the same time SDNDr of PC1 and phycocyanin Fill factor signaled non-random patterns of even spacing of traits (Fig. 3). PC1 and phycocyanin Fill, therefore, showed significant evidence for trait clustering and even dispersion, respectively, at the same time. Overall, however, standard effect sizes computed for our statistics were relatively small (Table S5), suggesting modest deviations from null model expectations.

## Discussion

### Spring Bloom Dynamics

In temperate lakes, spring blooms are commonly triggered by the transition from strong to weak vertical mixing and the associated onset of thermal stratification of the water column, due to heat flux increase [44], [45]. The bloom and the succession that follows are summarized by the broadly accepted Plankton Ecology Group (PEG) model [22], [23]. The original PEG model suggests that the main drivers of spring phytoplankton communities are physics (responding to the weather), grazing, and water chemistry [23]. Ciliates are the first herbivores appearing in spring and the first to graze on the phytoplankton community, and their important role in spring-bloom dynamics has become increasingly recognized [22], [46], [47]. Our data suggest ciliates were the main grazers of the phytoplankton community during the early spring-bloom, and were followed by multicellular larger zooplankton species [46], [47] (Copepods and Cladocera) that may have played a role as grazers after the ciliate bloom was over (Fig. 2B) [23], [46] [47]. Mixotrophic algae, such as some Chrysophytes, Cryptophytes and Dinophytes that are able to eat bacteria and smaller eukaryotic algae and were present in the spring phytoplankton community (Table S2), may have played a minor role in the grazing phase of the bloom [24]. The outcome of the grazing period was the dominance of *P. rubescens*, which is inedible, toxic [48], [49] and known to dominate the Lake Zurich phytoplankton community for extended periods of the year [48], [49] [50].

In our study, the descriptions of the phytoplankton spring bloom obtained by flow-cytometry and microscopy appeared to be similar. Cytobuoy-derived phytoplankton concentrations were similar to microscopic counts throughout the bloom period, except from the peak of phytoplankton abundance in which flow-cytometry counts exceeded microscopic counts by a factor of 2 (data not shown). Biovolume levels obtained by Cytobuoy analysis fall within the range expected for the spring community in lake Zurich [51], [52]. Previous work has highlighted how phytoplankton richness derived by using flow-cytometry based groups can be comparable with the total number of taxa detected by microscopy [27], [28].

In this study flow-cytometry derived richness appeared to deviate from species richness (Fig. 2C). Cytobuoy trait-based classes may include more than one species sharing similar physio-morphological characters. The fact that identity (and abundance) of trait-based groups may not reflect the identity (and abundance) of microscopically defined taxonomic groups was noted before [27]. The difference in microscopic and flow-cytometry descriptions of phytoplankton richness may be the result of automated measurements of cells not being able to recognize the full set of physio-morphological differences present in the natural community and used for taxonomic classification. On the other hand, the flow-cytometry description of phytoplankton turnover captured more dynamics than microscopic analysis (Fig. 2C–D). The difference in measures of richness and turnover may result from communities a) characterized by taxa sharing similar traits related to grazing-resistance or habitat-occupancy (as a consequence of selection) and b) rapidly changing though time [23], [24].

### Relationships between Size-related Traits and the Environment

Size (and shape) of cells and colonies represent “master” traits in phytoplankton, affecting many functions involved in metabolic acclimation, buoyancy, environmental tolerance and resistance to predators [53]. Previous work predicts that phytoplankton size would be under selection from grazing by meso- and macro-zooplankton [23], less from ciliates [54], by favoring larger (less easily edible) organisms, and from temperature and nutrients favoring smaller organisms (due to increased metabolism and more efficient surface/volume ratio, respectively) [18], [24], [53], [55].

In our study, we did not find any statistically significant link between phytoplankton size-related descriptors (such as length, number of cells per colony, and PC1) and abundance of ciliates using the fourth-corner test for environmental filtering (Table 2), which would be expected if ciliates acted as generalist grazers over the whole period of study (Table 1). Community-wide trait-distribution during phase 2 of the spring bloom succession, however, signaled significant even spacing of PC1 and size in the community, and reduced range of size (Table 1, Fig. 3). These patterns may be a signal of selection by multiple processes, such as mixed specialist and generalist grazers (Table 1) [4], [10], [15], [25].

Winter conditions, which pose major environmental constraints for the growth of plankton [23] (day one of the time series, Fig. 1), were characterized by environmental filtering on phytoplankton shape (PC1, Fig. 3). Both PC1 and size showed expected signals of selection by abiotic environmental filters (such as temperature and water chemistry, Table 2) in phase 3 of the bloom (Fig. 3). The relationships that emerge from the fourth-corner analysis between length, number of cells per colony and PC1 with temperature may reflect part of these abiotic environmental filters, and be indicative of acclimation processes: increase in temperature determining an increase in growth and size [55], and temperature-induced higher growth rates that can increase the number of cells per colony thereby increasing particle size [56]. The linkages with oxygen (positive) and conductivity (determined by carbonate and bicarbonate ions – negative) are probably due to coupled trends in phytoplankton growth during the period of study with high O_2_ evolution and bi-carbonate depletion through phytoplankton primary productivity (Fig. 1, Fig. 2).

### Relationships between FL-related Traits and the Environment

Parameters associated with FL signals relate to the concentration, distribution, and absorption efficiency of pigments, which impact on the operational photon yield of the photosynthetic apparatus and can be influenced by photoinhibition and quenching by packaging effects [57]–[59], disturbance or availability of light and nutrients [58]. In our study, changes in Fill factor of FL signals showed the most significant links to environmental fluctuations (Table 2).

The Fill factor represents a measure of pigment content and distribution within particles that, contrary to the other descriptors, is inherently independent from particle size [33]. This parameter denotes how much of the particle structure is filled with pigment and depends on particle shape, pigment content and distribution. Here, Fill factors for the yellow and orange channel showed a negative link to temperature, oxygen and abundance of ciliates in the fourth-corner test (Table 2), and a positive relationship with conductivity and PO4 (for α = 0.1, Table S3). Relationships between environmental variables and pigment fill may have been a consequence of acclimation to temperature, nutrients and light, although these can be complex [60] and species-specific [59]. It appears from our data that more pigment packaging (low fill factor, i.e. uneven distribution of pigments within particles) was linked to higher temperatures, phytoplankton growth and grazing pressure (Table 2).

Community wide trait analysis showed that FL signal distributions during phase 1 of the bloom signaled phenotypic clustering, a pattern consistent with selection by environmental filters on the distribution of these traits [11], [14] (Table 1). In plant communities, traits associated with leaf economics (including Chl-a content) have similarly been shown to be sensitive to environmental filtering processes [12]. In the grazing phase of the bloom, however, pigment Fill distributions were not distinguishable from null-model simulation (Fig. 3), indicating that possible selection by grazers was not strong on these traits.

### Correlation between Traits under Selection

**Figure 4:**
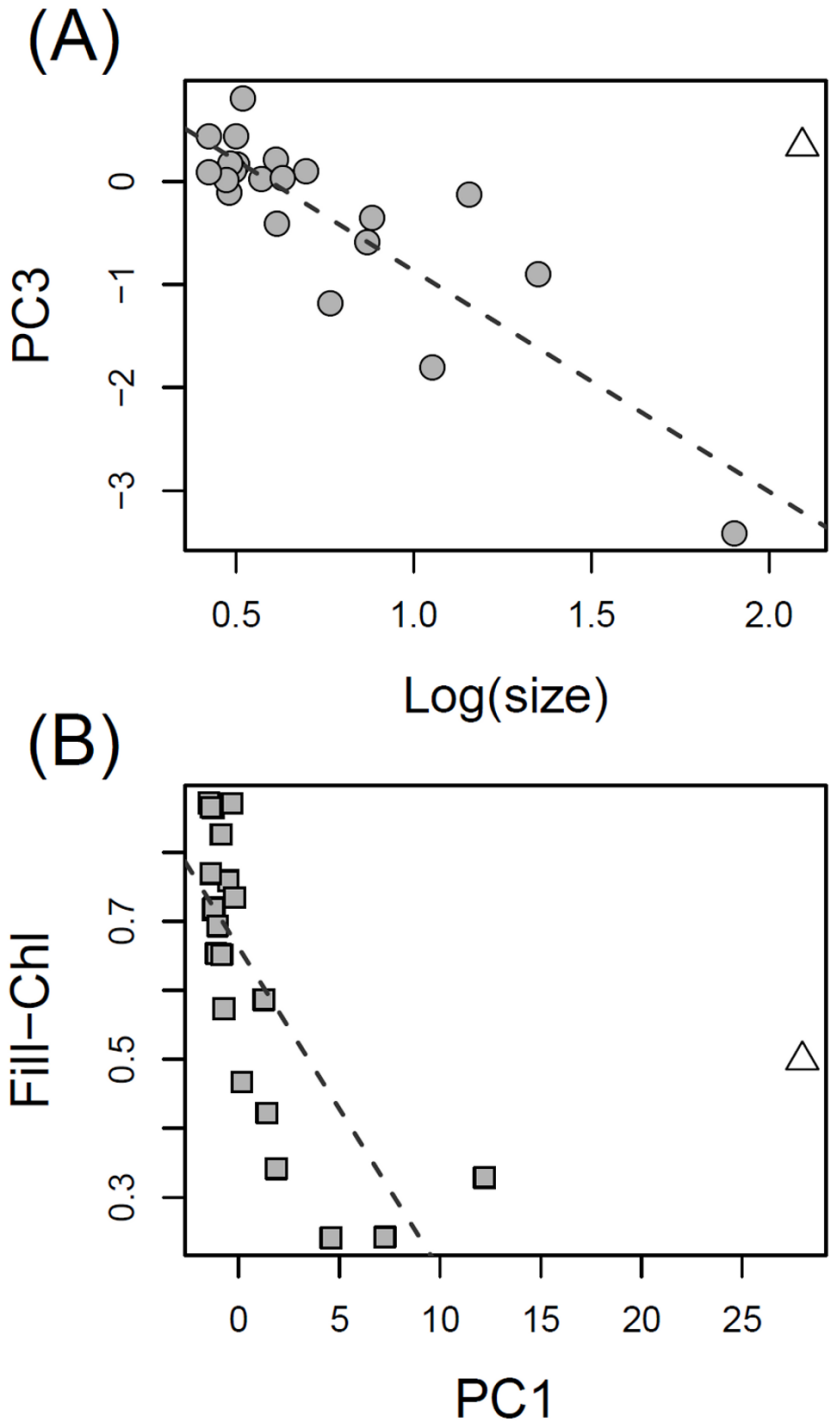
Correlations between average values of hypothesized habitat-occupancy and species-interaction traits for the 22 groups derived in this study by flow-cytometry. A) relationship between average size and PC3 (phytoplankton FL); B) relationship between PC1 (phytoplankton morphology) and Chl-a pigment fill. For PC loadings see Table S1. Dashed lines represent linear regression models (all fits and coefficients were significant at *p*<0.05). The triangular data point in A and B represents a group characterized by large *P. rubescens* filaments (data not shown), which appeared to be an outlier in all trait correlations and was excluded from the corresponding regression models.

In this study, we found weak relationships between traits and environmental variables (Table 2), and weak/sparse deviations of community wide trait distributions from random patterns (Fig. 3, Table S5). Our data, however, support the hypothesis that size-related traits were under selection by grazing in natural populations [22], [23], and that the Fill factor may have been linked to acclimation to abiotic environmental conditions. We suggest that the size and shape of phytoplankton cells or colonies may represent species-interaction traits, and pigment FL-related parameters may represent habitat-occupancy traits (Table 1). Average levels of size and PC1 of phytoplankton groups were inversely related to their mean FL and Chl-a Fill factor in our study (Fig. 4). We interpret these negative correlations as larger particles being less rich in pigments compared to smaller phytoplankton. Smaller cells, which have better SA/V ratio for nutrient uptake, may have been richer in active pigments to sustain higher growth rates, which would be necessary to support a population that suffers from high grazing pressure. Larger cells and colonies have more limited ability to access nutrients and (from our data) lower levels of active pigments, but are inherently more resistant to grazing and require lower growth rates to sustain populations. Advantages and trade-offs have been studied for some important phytoplankton physiological and morphological traits related to nutrient and light acquisition/utilization and predator avoidance, which impact on access to resources, growth, survival and reproduction [17], [18], [53]. Our data support laboratory studies highlighting the role of size and shape related traits for growth and resistance to grazing with evidence from natural phytoplankton communities, and suggest a tradeoff between environmental tolerance (in this case related to photosynthetic performance and acclimation to different depths) and grazing resistance traits (Fig. 4).

### Conclusions and Outlook

In this study we presented an approach to study phytoplankton biodiversity based on scanning flow-cytometry, allowing to measure individually expressed phytoplankton physio-morphological traits and to test their response to environmental changes. Our approach is different from previously employed trait-based approaches (relying on species-averaged traits, qualitative information and/or laboratory acclimated populations) since it allows to classify individually scanned particles into categories that reflected the physiological and morphological state of the organisms in their natural environment [29]. It should be noted, however, that Cytobuoy data lack physiological traits directly associated with nutrient uptake and metabolism, as well as general behavioral and life-history traits which may be important in understanding ecological interactions and community dynamics. Integration of scanning flow-cytometry data with additional trait information can broaden the list of possible traits under ecological selection, and may allow finer studies in the future targeting coexistence mechanisms, such as testing for niche and neutral processes in natural phytoplankton communities.

Overall, during a dynamic spring bloom succession of producers and their grazers, we detected evidence for selection on a set of phytoplankton physio-morphological traits in natural communities. Our data support the hypothesis that size- and morphology-related traits are those under the strongest selection from grazing and suggest that FL signals, and in particular pigment Fill, may be important traits linked to phytoplankton acclimation to the abiotic environment. A distinction of these traits into habitat differences and competitive ability differences (as in Table 1) may allow in the future a better understanding of species coexistence mechanisms [1], [3], [4]. Our data suggest a trade-offs among key competitive traits such as size, shape and coloniality (which influence grazing resistance), and active pigment levels, which relate to photosynthetic performance and acclimation to different habitats over the vertical structure of a deep lake.

Our study, however, has limitations and more work is needed to truly characterize Cytobuoy-derived traits. We relied on available tests that are based on trait-means per group, and recent evidence suggests that accounting for within-group trait variation increases the power of trait-based tests [12], [61]. Additionally, previous work has suggested that fourth-corner results and null model based trait dispersion analyses can be sensitive to a) low number of species, b) if the investigated species have wide environmental tolerance, or alternatively c) if data are affected by the presence of background random noise, which is expected in complex scenarios [14], [30]. All these factors may have affected our ability to detect and correctly interpret trait-patterns. An additional limitation of our study lays in analysis of a single bloom event, not replicated across seasons or over different years. Ecological events like spring blooms may be extremely context dependent and sensitive to the contingent weather and water conditions. Recent developments in automated aquatic ecosystem monitoring may aid in this direction, allowing to track changes in phytoplankton morpho-physiological categories and their traits under selection relative to their growth environment across seasons and years [27].

## Supporting Information

Table S1 **Factor loadings of PCA on Cytobuoy-derived parameters.**
(DOCX)

Table S2 **Microscopic counts (cells/mL) and taxonomic affiliation of Lake Zurich phytoplankton during the period of study.**
(DOCX)

Table S3 **Results of the two-step fourth corner analysis (p-values) performed on Cytobuoy-derived phytoplankton traits and environmental variables.**
(DOCX)

Table S4 **Results of trait-based tests (p-values) of community assembly for Cytobuoy-derived traits at each sampling date of our study period.**
(DOCX)

Table S5 **Results of trait-based tests (standardized effect sizes) of community assembly for Cytobuoy-derived traits at each sampling date of our study period.**
(DOCX)
