# Supplementary material for: Individual Cell Based Traits Obtained by Scanning Flow-Cytometry Show Selection by Biotic and Abiotic Environmental Factors during a Phytoplankton Spring Bloom
Source: PLoS One. 2013 Aug 12;8(8):e71677. doi: 10.1371/journal.pone.0071677 (PMC3741118; doi:10.1371/journal.pone.0071677)
Supplement: Table S1 — Factor loadings of PCA on Cytobuoy-derived parameters. (DOCX) [file pone.0071677.s001.docx]

**Table S1.** Factor loadings of PCA on Cytobuoy-derived parameters.

|  | *PC1* | *PC2* | *PC3* | *PC4* | *PC5* | *PC6* | *PC7* | *PC8* |
| --- | --- | --- | --- | --- | --- | --- | --- | --- |
| *Standard deviation* | 4.286 | 3.102 | 1.7531 | 1.6307 | 1.3828 | 1.2046 | 1.0392 | 1.0082 |
| *Proportion of variance* | 0.399 | 0.209 | 0.0668 | 0.0578 | 0.0416 | 0.0315 | 0.0235 | 0.0221 |
| *Cumulative proportion* | 0.399 | 0.609 | 0.6753 | 0.7331 | 0.7747 | 0.8063 | 0.8297 | 0.8518 |
| *Cytobuoy parameters* |  |  |  |  |  |  |  |  |
| Length.FWS | 0.207 | 0.118 | -0.001 | 0.044 | 0.072 | -0.020 | 0.129 | 0.031 |
| Length.SWS | 0.172 | 0.087 | 0.147 | 0.052 | -0.165 | 0.091 | 0.164 | 0.180 |
| Length.FL.Yellow | 0.199 | 0.126 | -0.034 | 0.040 | 0.127 | -0.096 | 0.030 | -0.047 |
| Length.FL.Orange | 0.194 | 0.122 | -0.068 | 0.035 | 0.163 | -0.103 | 0.029 | -0.039 |
| Length.FL.Red | 0.197 | 0.122 | -0.036 | 0.037 | 0.113 | -0.080 | 0.043 | 0.013 |
| Total.FWS | 0.137 | 0.066 | 0.221 | 0.079 | -0.283 | 0.066 | 0.212 | 0.424 |
| Total.SWS | 0.202 | 0.085 | 0.085 | 0.064 | -0.058 | -0.011 | 0.156 | 0.198 |
| Total.FL.Yellow | 0.166 | 0.041 | -0.294 | -0.043 | 0.024 | -0.133 | 0.037 | 0.082 |
| Total.FL.Orange | 0.165 | 0.076 | -0.247 | -0.010 | 0.235 | -0.088 | 0.004 | 0.180 |
| Total.FL.Red | 0.183 | 0.053 | -0.224 | -0.009 | 0.218 | -0.004 | -0.084 | 0.157 |
| Max.FWS | 0.135 | -0.209 | 0.072 | 0.042 | -0.115 | 0.158 | -0.116 | 0.002 |
| Max.SWS | 0.160 | -0.162 | 0.056 | -0.047 | 0.001 | 0.006 | -0.198 | -0.223 |
| Max.FL.Yellow | 0.058 | -0.083 | -0.287 | -0.126 | -0.450 | -0.186 | 0.125 | -0.163 |
| Max.FL.Orange | 0.119 | -0.115 | -0.275 | -0.165 | -0.185 | 0.096 | -0.220 | 0.123 |
| Max.FL.Red | 0.111 | -0.182 | -0.098 | -0.087 | -0.030 | 0.302 | -0.377 | 0.140 |
| Avg.FWS | 0.156 | -0.125 | 0.122 | 0.129 | -0.175 | 0.163 | 0.084 | 0.237 |
| Avg.SWS | 0.191 | -0.111 | 0.064 | -0.005 | -0.018 | 0.025 | -0.102 | -0.105 |
| Avg.FL.Yellow | 0.042 | -0.048 | -0.307 | -0.123 | -0.451 | -0.248 | 0.191 | -0.142 |
| Avg.FL.Orange | 0.150 | -0.037 | -0.345 | -0.116 | -0.068 | -0.027 | -0.022 | 0.153 |
| Avg.FL.Red | 0.135 | -0.144 | -0.153 | -0.023 | 0.073 | 0.277 | -0.259 | 0.223 |
| Inertia.FWS | -0.028 | 0.237 | -0.030 | -0.250 | -0.051 | 0.322 | -0.013 | -0.159 |
| Inertia.SWS | -0.011 | 0.227 | -0.020 | -0.238 | 0.000 | 0.304 | 0.196 | -0.118 |
| Inertia.FL.Yellow | -0.071 | 0.224 | 0.046 | -0.092 | -0.012 | -0.204 | -0.218 | 0.092 |
| Inertia.FL.Orange | -0.067 | 0.231 | 0.055 | -0.083 | -0.055 | -0.202 | -0.325 | 0.105 |
| Inertia.FL.Red | -0.057 | 0.234 | 0.013 | -0.179 | -0.088 | -0.020 | -0.218 | -0.007 |
| CG.FWS | 0.221 | 0.071 | 0.043 | 0.047 | 0.010 | -0.014 | 0.067 | -0.004 |
| CG.SWS | 0.222 | 0.067 | 0.053 | 0.046 | -0.003 | -0.008 | 0.059 | -0.005 |
| CG.FL.Yellow | 0.222 | 0.064 | -0.006 | 0.037 | 0.075 | -0.034 | 0.009 | -0.099 |
| CG.FL.Orange | 0.221 | 0.065 | -0.007 | 0.036 | 0.078 | -0.034 | 0.011 | -0.102 |
| CG.FL.Red | 0.221 | 0.066 | -0.001 | 0.039 | 0.069 | -0.033 | 0.014 | -0.095 |
| Fill.FWS | -0.052 | 0.250 | -0.075 | -0.171 | -0.037 | 0.303 | 0.066 | -0.022 |
| Fill.SWS | -0.054 | 0.249 | -0.068 | -0.150 | -0.013 | 0.292 | 0.227 | 0.015 |
| Fill.FL.Yellow | -0.093 | 0.243 | -0.048 | 0.035 | 0.027 | -0.156 | -0.033 | 0.173 |
| Fill.FL.Orange | -0.090 | 0.252 | -0.028 | 0.013 | -0.011 | -0.172 | -0.163 | 0.189 |
| Fill.FL.Red | -0.080 | 0.253 | -0.060 | -0.076 | -0.047 | -0.016 | -0.101 | 0.100 |
| Asymm.FWS | 0.061 | -0.152 | 0.173 | -0.295 | 0.086 | -0.204 | 0.046 | 0.042 |
| Asymm.SWS | 0.060 | -0.139 | 0.180 | -0.354 | 0.070 | -0.158 | -0.012 | 0.004 |
| Asymm.FL.Yellow | 0.033 | -0.080 | 0.106 | -0.355 | 0.084 | -0.040 | 0.081 | 0.184 |
| Asymm.FL.Orange | 0.047 | -0.109 | 0.125 | -0.383 | 0.095 | -0.073 | 0.090 | 0.117 |
| Asymm.FL.Red | 0.044 | -0.111 | 0.135 | -0.383 | 0.116 | -0.081 | 0.126 | 0.059 |
| X.Cells.FWS | 0.181 | 0.099 | 0.000 | -0.090 | 0.074 | 0.086 | -0.072 | -0.328 |
| X.Cells.SWS | 0.206 | 0.055 | 0.061 | -0.032 | 0.019 | 0.035 | -0.018 | -0.227 |
| X.Cells.FL.Yellow | 0.147 | 0.134 | 0.204 | 0.012 | -0.158 | -0.056 | -0.152 | -0.161 |
| X.Cells.FL.Orange | 0.142 | 0.129 | 0.234 | -0.006 | -0.236 | -0.053 | -0.253 | -0.053 |
| X.Cells.FL.Red | 0.140 | 0.110 | 0.224 | -0.038 | -0.256 | 0.004 | -0.163 | 0.033 |
