# Supplementary material for: Individual Cell Based Traits Obtained by Scanning Flow-Cytometry Show Selection by Biotic and Abiotic Environmental Factors during a Phytoplankton Spring Bloom
Source: PLoS One. 2013 Aug 12;8(8):e71677. doi: 10.1371/journal.pone.0071677 (PMC3741118; doi:10.1371/journal.pone.0071677)
Supplement: Table S2 — Microscopic counts (cells/mL) and taxonomic affiliation of Lake Zurich phytoplankton during the period of study. (DOCX) [file pone.0071677.s002.docx]

**Table S2.** Microscopic counts (cells / mL) and taxonomic affiliation of Lake Zurich phytoplankton during the period of study (between March and May 2009).

|  | *Date* | *23/03* | *30/03* | *03/04* | *07/04* | *09/04* | *14/04* | *15/04* | *17/04* | *20/04* | *22/04* | *24/04* | *28/04* | *30/04* | *04/05* | *06/05* |
| --- | --- | --- | --- | --- | --- | --- | --- | --- | --- | --- | --- | --- | --- | --- | --- | --- |
|  | *Day* | *1* | *8* | *12* | *16* | *18* | *23* | *24* | *26* | *29* | *31* | *33* | *37* | *39* | *43* | *45* |
| *Class* | *Species* |  |  |  |  |  |  |  |  |  |  |  |  |  |  |  |
| Bacillariophyceae | *Asterionella formosa* | 140 | 100 | 107 | 140 | 260 | 200 | 160 | 110 | 58 | 39 | 15 | 4.3 | 5.4 | 1.7 | 0 |
| Bacillariophyceae | *Cyclotella* sp. small | 25 | 35 | 59 | 130 | 370 | 280 | 210 | 340 | 350 | 160 | 160 | 170 | 230 | 280 | 270 |
| Bacillariophyceae | *Cyclotella* sp. medium | 49 | 29 | 35 | 110 | 230 | 170 | 160 | 130 | 130 | 120 | 62 | 21 | 86 | 100 | 72 |
| Bacillariophyceae | *Diatoma elongate* | 0 | 0 | 0 | 10 | 0 | 4.5 | 4 | 26 | 7.9 | 28 | 4.5 | 5.8 | 6.7 | 0 | 3.4 |
| Bacillariophyceae | *Fragilaria acus acus* | 1.3 | 0.56 | 0 | 4.3 | 12 | 58 | 55 | 38 | 78 | 87 | 50 | 36 | 24 | 5.6 | 5.8 |
| Bacillariophyceae | *Fragilaria crotonensis* | 13 | 79 | 21 | 39 | 94 | 150 | 120 | 110 | 190 | 490 | 82 | 110 | 100 | 1.1 | 17 |
| Bacillariophyceae | *Fragilaria ulna* | 17 | 5.6 | 15 | 19 | 48 | 88 | 90 | 94 | 120 | 120 | 85 | 78 | 88 | 22 | 25 |
| Bacillariophyceae | *Melosira* sp. | 13 | 48 | 25 | 180 | 18 | 25 | 0 | 16 | 5.6 | 0 | 6.7 | 0 | 0 | 0 | 0 |
| Bacillariophyceae | *Navicula* sp. | 0 | 1.7 | 0 | 0 | 0 | 0 | 0 | 0 | 0 | 0 | 0 | 0 | 0 | 0 | 0 |
| Bacillariophyceae | *Stephanodiscus* sp. | 12 | 10 | 3.5 | 0 | 0 | 0 | 0 | 0 | 0 | 0 | 0 | 0 | 0 | 0 | 0 |
| Bacillariophyceae | *Tabellaria fenestrate* | 0 | 0 | 0 | 0 | 0 | 0 | 3.4 | 4.5 | 9 | 4 | 4 | 8.7 | 1.3 | 0 | 3.9 |
| Charophyceae | *Elakatothrix* sp. | 0 | 0 | 0 | 0 | 0 | 0 | 0.37 | 1.2 | 0 | 0 | 0 | 1 | 0.37 | 72 | 6.7 |
| Chlorophyceae | *Phacotus lendneri* | 0 | 0 | 0 | 0 | 0.77 | 0 | 0 | 0 | 0 | 0 | 0 | 0 | 0 | 0 | 0 |
| Chrysophyceae | *Dinobryon* sp. | 0 | 0 | 0 | 0 | 0 | 200 | 260 | 230 | 150 | 410 | 100 | 64 | 85 | 59 | 16 |
| Chrysophyceae | *diverse Chrysophyceae* | 51 | 30 | 29 | 110 | 280 | 550 | 170 | 460 | 300 | 210 | 360 | 100 | 240 | 480 | 340 |
| Chrysophyceae | *Erkenia* sp. | 37 | 12 | 10 | 320 | 410 | 1100 | 250 | 3100 | 260 | 55 | 152 | 190 | 48 | 590 | 28 |
| Chrysophyceae | *Mallomonas* sp. | 0 | 0.58 | 0 | 0 | 0 | 1.7 | 17 | 0 | 0 | 0 | 4 | 0 | 0 | 0 | 0 |
| Cryptophyceae | *Cryptomonas* sp. | 8.3 | 9 | 9.2 | 58 | 140 | 35 | 36 | 24 | 32 | 12 | 46 | 4.6 | 14 | 3.5 | 9.2 |
| Cryptophyceae | *Rhodomonas lacustris*  **Table S2. Continuing.** | 410 | 81 | 410 | 2600 | 7600 | 1900 | 2200 | 810 | 1300 | 950 | 260 | 800 | 650 | 790 | 170 |
| Cryptophyceae | *Rhodomonas lacustris as lens* | 97 | 21 | 150 | 1400 | 5200 | 2800 | 3600 | 1400 | 2400 | 2300 | 190 | 150 | 810 | 360 | 640 |
| Cyanobacteria | *Aphanizomenon* sp. | 0 | 0 | 0 | 14 | 0 | 0 | 0 | 0 | 0 | 0 | 11 | 0 | 17 | 56 | 0 |
| Cyanobacteria | *Planktothrix rubescens* | 4500 | 3800 | 3200 | 2000 | 2400 | 2400 | 2200 | 2800 | 4700 | 3800 | 1800 | 3300 | 8500 | 9600 | 19000 |
| Dinophyceae | *Ceratium* sp. | 0 | 0 | 0 | 0 | 0 | 0.2 | 0.19 | 0 | 0 | 0 | 0 | 0 | 0 | 0 | 0 |
| Dinophyceae | *Gymnodinium helveticum* | 0 | 1.7 | 4.2 | 2.9 | 2.1 | 3.4 | 7.9 | 7.2 | 2.7 | 3.3 | 6.7 | 5.2 | 6.7 | 3 | 5.5 |
| Dinophyceae | *Gymnodinium lantzschii* | 4.6 | 3.5 | 12 | 17 | 36 | 32 | 52 | 18 | 35 | 26 | 20 | 17 | 18 | 3.5 | 3.5 |
| Dinophyceae | *Peridinium* sp. | 0 | 0 | 0 | 0 | 0.42 | 0.4 | 0 | 0 | 0 | 0 | 0 | 0 | 0 | 0 | 0 |
| Katablepharid. | *Katablepharis* sp. | 17 | 0 | 0 | 0 | 0 | 190 | 83 | 90 | 140 | 62 | 0 | 0 | 55 | 110 | 83 |
| Zygnematoph. | *Closterium aciculare* | 0 | 0 | 0 | 0 | 0 | 0 | 0 | 0 | 0 | 0 | 0 | 0.18 | 0 | 0 | 0 |
| Zygnematoph. | *Cosmarium depressum* | 0 | 0 | 0 | 0 | 0 | 0 | 0.19 | 0 | 0 | 0.3 | 0.56 | 0 | 0.7 | 0.4 | 0.76 |
|  | *TOTAL* | 5395 | 4267 | 4090 | 7154 | 17101 | 10188 | 9680 | 9809 | 10268 | 8876 | 3419 | 5066 | 10986 | 12538 | 20700 |
