# Supplementary material for: Individual Cell Based Traits Obtained by Scanning Flow-Cytometry Show Selection by Biotic and Abiotic Environmental Factors during a Phytoplankton Spring Bloom
Source: PLoS One. 2013 Aug 12;8(8):e71677. doi: 10.1371/journal.pone.0071677 (PMC3741118; doi:10.1371/journal.pone.0071677)
Supplement: Table S3 — Results of the two-step fourth corner analysis (p-values) performed on Cytobuoy-derived phytoplankton traits and environmental variables. (DOCX) [file pone.0071677.s003.docx]

**Table S3.** Results of the two-step fourth corner analysis (p-values) performed on Cytobuoy-derived phytoplankton traits and environmental variables. Significant correlations at *p* > 0.05 are highlighted in bold (n = 22-groups x 15-sites x 15-traits).

| *Cytobuoy-derived traits* | *Temperature* | *Conductivity* | *Oxygen%* | *DOC* | *PO4* | *NO3* | *Ciliates* |
| --- | --- | --- | --- | --- | --- | --- | --- |
| PC1 | **0.033** | **0.018** | **0.033** | 0.306 | 0.172 | 0.237 | 0.127 |
| Length.SWS | **0.034** | **0.028** | **0.028** | 0.353 | 0.223 | 0.291 | 0.166 |
| Total.FL.Yellow | 0.095 | **0.035** | 0.081 | 0.448 | 0.300 | 0.410 | 0.269 |
| Total.FL.Orange | 0.226 | 0.091 | 0.194 | 0.543 | 0.400 | 0.524 | 0.413 |
| Total.FL.Red | 0.118 | **0.046** | 0.090 | 0.451 | 0.321 | 0.431 | 0.291 |
| Max.FL.Yellow | 0.078 | **0.046** | 0.080 | 0.384 | 0.198 | 0.215 | 0.139 |
| Max.FL.Orange | 0.061 | **0.025** | **0.049** | 0.346 | 0.231 | 0.275 | 0.154 |
| Max.FL.Red | 0.074 | **0.037** | 0.064 | 0.282 | 0.106 | 0.156 | 0.088 |
| Fill.FL.Yellow | 0.052 | **0.014** | **0.031** | 0.161 | 0.080 | 0.082 | **0.029** |
| Fill.FL.Orange | **0.041** | **0.017** | **0.032** | 0.163 | 0.073 | 0.102 | **0.041** |
| Fill.FL.Red | 0.089 | 0.052 | 0.057 | 0.156 | 0.092 | 0.105 | 0.071 |
| Num.Cells.SWS | **0.021** | **0.025** | **0.017** | 0.298 | 0.159 | 0.216 | 0.118 |
| Num.Peaks.FL.Yellow | 0.056 | **0.020** | **0.045** | 0.406 | 0.328 | 0.274 | 0.177 |
| Num. Peaks.FL.Orange | 0.082 | **0.018** | 0.062 | 0.406 | 0.324 | 0.275 | 0.186 |
| Num. Peaks.FL.Red | 0.067 | **0.026** | 0.059 | 0.356 | 0.260 | 0.277 | 0.155 |
