# Supplementary material for: Individual Cell Based Traits Obtained by Scanning Flow-Cytometry Show Selection by Biotic and Abiotic Environmental Factors during a Phytoplankton Spring Bloom
Source: PLoS One. 2013 Aug 12;8(8):e71677. doi: 10.1371/journal.pone.0071677 (PMC3741118; doi:10.1371/journal.pone.0071677)
Supplement: Table S4 — Results of trait-based tests (p-values) of community assembly for Cytobuoy-derived traits at each sampling date of our study period. (DOCX) [file pone.0071677.s004.docx]

**Table S4.** Results of trait-based tests (p-values) of community assembly for Cytobuoy-derived parameters at each sampling date of our study period. Mean and range test for environmental filtering, SDNDr for even spacing of traits. Tests significant at p < 0.05 are highlighted in bold. Grey shaded cells emphasise the period of ciliate grazing.

| *Trait and date* | *Day* | *Mean* | *Range* | *SDNDr* |
| --- | --- | --- | --- | --- |
| *PC1^a^* |  |  |  |  |
| 23.03.09 | 1 | **0.020** | **0.043** | 0.170 |
| 30.03.09 | 8 | 0.062 | 0.063 | 0.448 |
| 03.04.09 | 12 | 0.825 | 0.386 | 0.280 |
| 07.04.09 | 16 | 0.505 | 0.323 | 0.510 |
| 09.04.09 | 18 | 0.354 | 0.185 | **0.023** |
| 14.04.09 | 23 | 0.280 | 0.121 | **0.005** |
| 15.04.09 | 24 | 0.406 | 0.198 | **0.016** |
| 17.04.09 | 26 | 0.320 | 0.141 | 0.067 |
| 20.04.09 | 29 | 0.344 | 0.130 | 0.064 |
| 22.04.09 | 31 | 0.292 | 0.083 | 0.114 |
| 24.04.09 | 33 | 0.300 | 0.185 | 0.229 |
| 28.04.09 | 37 | 0.062 | 0.055 | 0.196 |
| 30.04.09 | 39 | **0.016** | **0.004** | **0.000** |
| 04.05.09 | 43 | **0.024** | **0.007** | **0.002** |
| 06.05.09 | 45 | 0.895 | 0.494 | 0.913 |
| *Length.SWS^b^* |  |  |  |  |
| 23.03.09 | 1 | 0.404 | 0.301 | 0.950 |
| 30.03.09 | 8 | 0.106 | 0.167 | 0.815 |
| 03.04.09 | 12 | 0.613 | 0.372 | 0.491 |
| 07.04.09 | 16 | 0.202 | 0.304 | 0.803 |
| 09.04.09 | 18 | 0.346 | 0.144 | **0.047** |
| 14.04.09 | 23 | 0.330 | 0.091 | **0.000** |
| 15.04.09 | 24 | 0.655 | 0.258 | 0.222 |
| 17.04.09 | 26 | 0.080 | **0.031** | **0.001** |
| 20.04.09 | 29 | 0.074 | **0.047** | **0.004** |
| 22.04.09 | 31 | 0.166 | 0.062 | **0.016** |
| 24.04.09 | 33 | 0.082 | 0.088 | 0.166 |
| 28.04.09 | 37 | 0.060 | **0.034** | 0.059 |
| 30.04.09 | 39 | **0.006** | **0.004** | 0.137 |
| 04.05.09 | 43 | **0.002** | **0.003** | 0.156 |
| 06.05.09 | 45 | 0.456 | 0.706 | 0.925 |
| *Fill.FL.Red^c^* |  |  |  |  |
| 23.03.09 | 1 | **0.000** | **0.002** | 0.852 |
| 30.03.09 | 8 | 0.086 | **0.040** | 0.165 |
| 03.04.09 | 12 | 0.667 | 0.812 | 0.708 |
| 07.04.09 | 16 | 0.973 | 0.678 | 0.093 |
| 09.04.09 | 18 | 0.807 | 0.770 | 0.080 |
| 14.04.09 | 23 | 0.833 | 0.688 | 0.157 |
| 15.04.09 | 24 | 0.965 | 0.640 | 0.054 |
| 17.04.09 | 26 | 0.751 | 0.189 | 0.093 |
| 20.04.09 | 29 | 0.753 | 0.189 | 0.111 |
| 22.04.09 | 31 | 0.759 | 0.300 | 0.204 |
| 24.04.09 | 33 | 0.995 | 0.242 | 0.104 |
| 28.04.09 | 37 | 0.364 | 0.095 | 0.427 |
| 30.04.09 | 39 | 0.276 | **0.025** | 0.333 |
| 04.05.09 | 43 | 0.290 | **0.029** | 0.356 |
| 06.05.09 | 45 | 0.999 | 0.271 | 0.057 |
| *Fill.FL.Orange^d^* |  |  |  |  |
| 23.03.09 | 1 | **0.008** | **0.013** | 0.910 |
| 30.03.09 | 8 | **0.006** | **0.007** | 0.633 |
| 03.04.09 | 12 | 0.811 | 0.941 | 0.851 |
| 07.04.09 | 16 | 0.563 | 0.689 | 0.911 |
| 09.04.09 | 18 | 0.721 | 0.735 | 0.815 |
| 14.04.09 | 23 | 0.597 | 0.658 | 0.539 |
| 15.04.09 | 24 | 0.807 | 0.628 | 0.210 |
| 17.04.09 | 26 | 0.869 | 0.165 | 0.200 |
| 20.04.09 | 29 | 0.873 | 0.144 | 0.186 |
| 22.04.09 | 31 | 0.963 | 0.313 | 0.275 |
| 24.04.09 | 33 | 0.268 | 0.252 | 0.738 |
| 28.04.09 | 37 | 0.350 | 0.051 | 0.212 |
| 30.04.09 | 39 | 0.208 | **0.046** | **0.018** |
| 04.05.09 | 43 | 0.180 | **0.041** | **0.013** |
| 06.05.09 | 45 | 0.941 | 0.575 | 0.225 |

^a^ First principal component of Cytobuoy-derived phytoplankton traits (Table S1).

^b^ Size of phytoplankton particles.

^c^ Chl-a particle fill (see Methods and Table 2).

^d^ Phycocyanin particle fill (see Methods and Table 2).
