# Supplementary material for: Individual Cell Based Traits Obtained by Scanning Flow-Cytometry Show Selection by Biotic and Abiotic Environmental Factors during a Phytoplankton Spring Bloom
Source: PLoS One. 2013 Aug 12;8(8):e71677. doi: 10.1371/journal.pone.0071677 (PMC3741118; doi:10.1371/journal.pone.0071677)
Supplement: Table S5 — Results of trait-based tests (standardized effect sizes) of community assembly for Cytobuoy-derived traits at each sampling date of our study period. (DOCX) [file pone.0071677.s005.docx]

**Table S5.** Results of trait-based tests (standardized effect sizes) of community assembly for Cytobuoy-derived parameters at each sampling date of our study period. Mean and range test for environmental filtering, SDNDr for even spacing of traits. Positive and negative values indicate an increase or decrease, respectively, in the observed metric compared to null model generated random expectations. Tests significant at *p* < 0.05 are highlighted in bold. Grey shaded cells emphasize the period of herbivore ciliate grazing.

| *Trait and date* | *Day* | *Mean* | *Range* | *SDNDr* |
| --- | --- | --- | --- | --- |
| *PC1^a^* |  |  |  |  |
| 23.03.09 | 1 | **-0.987** | **-0.905** | -0.947 |
| 30.03.09 | 8 | -1.218 | -1.148 | -0.258 |
| 03.04.09 | 12 | -0.494 | -0.517 | -0.694 |
| 07.04.09 | 16 | -0.816 | -0.739 | -0.162 |
| 09.04.09 | 18 | -0.946 | -1.082 | **-1.522** |
| 14.04.09 | 23 | -1.101 | -1.368 | **-1.653** |
| 15.04.09 | 24 | -0.856 | -1.064 | **-1.573** |
| 17.04.09 | 26 | -0.920 | -1.095 | -1.532 |
| 20.04.09 | 29 | -0.959 | -1.103 | -1.532 |
| 22.04.09 | 31 | -1.056 | -1.275 | -1.258 |
| 24.04.09 | 33 | -0.836 | -0.817 | -0.809 |
| 28.04.09 | 37 | -1.365 | -1.451 | -0.897 |
| 30.04.09 | 39 | **-1.300** | **-1.255** | **-2.489** |
| 04.05.09 | 43 | **-1.228** | **-1.250** | **-2.501** |
| 06.05.09 | 45 | -0.238 | 0.863 | 1.530 |
| *Length.SWS^b^* |  |  |  |  |
| 23.03.09 | 1 | -0.899 | -0.750 | 1.621 |
| 30.03.09 | 8 | -1.477 | -1.123 | 0.980 |
| 03.04.09 | 12 | -0.663 | -0.539 | -0.104 |
| 07.04.09 | 16 | -1.234 | -0.760 | 0.737 |
| 09.04.09 | 18 | -0.940 | -1.433 | **-1.422** |
| 14.04.09 | 23 | -1.025 | -1.805 | **-2.006** |
| 15.04.09 | 24 | -0.511 | -0.033 | -0.845 |
| 17.04.09 | 26 | -1.479 | **-1.760** | **-2.149** |
| 20.04.09 | 29 | -1.497 | **-1.725** | **-2.113** |
| 22.04.09 | 31 | -1.407 | -1.849 | **-1.818** |
| 24.04.09 | 33 | -1.290 | -1.192 | -0.997 |
| 28.04.09 | 37 | -1.742 | **-2.105** | -1.366 |
| 30.04.09 | 39 | **-1.833** | **-1.888** | -1.088 |
| 04.05.09 | 43 | **-1.829** | **-1.897** | -1.034 |
| 06.05.09 | 45 | -0.785 | 0.668 | 1.542 |
| *Fill.FL.Red^c^* |  |  |  |  |
| 23.03.09 | 1 | **2.010** | **-2.033** | 1.039 |
| 30.03.09 | 8 | 1.579 | **-2.257** | -0.988 |
| 03.04.09 | 12 | -0.384 | 1.169 | 0.522 |
| 07.04.09 | 16 | 0.072 | 0.734 | -1.156 |
| 09.04.09 | 18 | -0.192 | 0.590 | -1.133 |
| 14.04.09 | 23 | 0.252 | 0.470 | -0.871 |
| 15.04.09 | 24 | 0.079 | 0.394 | -1.064 |
| 17.04.09 | 26 | -0.307 | -1.080 | -1.199 |
| 20.04.09 | 29 | -0.304 | -1.128 | -1.154 |
| 22.04.09 | 31 | -0.294 | -0.026 | -0.839 |
| 24.04.09 | 33 | 0.093 | -0.915 | -1.366 |
| 28.04.09 | 37 | 0.922 | -1.237 | -0.351 |
| 30.04.09 | 39 | 1.052 | **-2.386** | -0.526 |
| 04.05.09 | 43 | 1.070 | **-2.381** | -0.481 |
| 06.05.09 | 45 | 0.089 | 0.454 | -1.175 |
| *Fill.FL.Orange^d^* |  |  |  |  |
| 23.03.09 | 1 | **1.938** | **-2.201** | 1.480 |
| 30.03.09 | 8 | **2.324** | **-3.111** | 0.168 |
| 03.04.09 | 12 | 0.343 | 1.191 | 1.104 |
| 07.04.09 | 16 | 0.620 | 0.746 | 1.459 |
| 09.04.09 | 18 | 0.424 | 0.579 | 0.616 |
| 14.04.09 | 23 | 0.561 | 0.450 | -0.009 |
| 15.04.09 | 24 | 0.246 | 0.402 | -0.807 |
| 17.04.09 | 26 | 0.248 | -0.821 | -0.815 |
| 20.04.09 | 29 | 0.213 | -0.892 | -0.845 |
| 22.04.09 | 31 | -0.043 | 0.513 | -0.606 |
| 24.04.09 | 33 | 1.146 | -0.862 | 0.655 |
| 28.04.09 | 37 | 0.949 | -1.554 | -0.754 |
| 30.04.09 | 39 | 1.221 | **-1.961** | **-1.611** |
| 04.05.09 | 43 | 1.258 | **-1.966** | **-1.635** |
| 06.05.09 | 45 | 0.120 | 0.466 | -0.707 |

^a^ First principal component of Cytobuoy-derived phytoplankton traits (Table S1).

^b^ Size of phytoplankton particles.

^c^ Chl-a particle fill (see Methods and Table 2).

^d^ Phycocyanin particle fill (see Methods and Table 2).
